# High Resolution Mapping of *Rph*_*MBR*1012_ Conferring Resistance to *Puccinia hordei* in Barley (*Hordeum vulgare* L.)

**DOI:** 10.3389/fpls.2019.00640

**Published:** 2019-05-22

**Authors:** Leila Fazlikhani, Jens Keilwagen, Doris Kopahnke, Holger Deising, Frank Ordon, Dragan Perovic

**Affiliations:** ^1^Institute for Resistance Research and Stress Tolerance, Federal Research Centre for Cultivated Plants, Julius Kühn-Institute (JKI), Quedlinburg, Germany; ^2^Department of Phytopathology and Plant Protection, Institute of Agricultural and Nutrition Sciences, Faculty of Natural Sciences III, Martin Luther University of Halle-Wittenberg, Halle, Germany; ^3^Institute for Biosafety in Plant Biotechnology, Federal Research Centre for Cultivated Plants, Julius Kühn-Institute (JKI), Quedlinburg, Germany

**Keywords:** barley, leaf rust resistance gene *Rph*_*MBR*1012_, positional isolation, GBS, Infinium 50K

## Abstract

Isolation of disease resistance genes in barley was hampered by the large genome size, but has become easy due to the availability of the reference genome sequence. During the last years, many genomic resources, e.g., the Illumina 9K iSelect, the 50K Infinium arrays, the Barley Genome Zipper, POPSEQ, and genotyping by sequencing (GBS), were developed that enable enhanced gene isolation in combination with the barley genome sequence. In the present study, we developed a fine map of the barley leaf rust resistance gene *Rph*_*MBR*1012_. 537 segmental homozygous recombinant inbred lines (RILs) derived from 4775 F_2_-plants were used to construct a high-resolution mapping population (HRMP). The Barley Genome Zipper, the 9K iSelect chip, the 50K Infinium chip and GBS were used to develop 56 molecular markers located in the target interval of 8 cM. This interval was narrowed down to about 0.07 cM corresponding to 0.44 Mb of the barley reference genome. Eleven low-confidence and 18 high-confidence genes were identified in this interval. Five of these are putative disease resistance genes and were subjected to allele-specific sequencing. In addition, comparison of the genetic map and the reference genome revealed an inversion of 1.34 Mb located distally to the resistance locus. In conclusion, the barley reference sequence and the respective gene annotation delivered detailed information about the physical size of the target interval, the genes located in the target interval and facilitated the efficient development of molecular markers for marker-assisted selection for *Rph_MBR1012._*

## Introduction

Leaf rust of barley is a serious disease caused by the biotrophic fungus *Puccinia hordei* Otth., which, under favorable conditions, may cause yield losses of up to 62% (Park et al., 2015), while in general loses are about 15–25% (Whelan et al., 1997). Symptoms of leaf rust vary from small chlorotic flecks to large orange-brown pustules of up to 0.5 mm in size, often surrounded by green islands (Clifford, 1985). Although several resistance genes in barley have been identified, the major challenge in control of barley leaf rust is the breakdown of resistance caused by mutations in effector (avirulence) genes of the pathogen, leading to occurrence of new virulent races on previously resistant plant cultivars in a short period of time (Park, 2003). Therefore, to combat leaf rust epidemics caused by newly occurring/generated virulent races and to achieve a sustainable disease control, the employment of new resistance genes using functional molecular markers in breeding schemes as well as the isolation of known ones in order to get detailed information on the structure and function is of prime importance. Furthermore, isolation of known resistance genes is a prerequisite that allow an efficient allele mining of genetic resources (Li et al., 2016) as well as allele editing, e.g., by CRISPR/Cas9 (Wang et al., 2014).

Since the first genetic study on leaf rust resistance (Waterhouse, 1927), 25 *Rph* (Resistance to *P. hordei*) genes have been mapped in barley (Kavanagh et al., 2017). Among them, two genes, namely *Rph20* and *Rph23*, mediate an adult plant resistance (APR) (Hickey et al., 2011; Singh et al., 2015), while the remaining 23 (*Rph1* to *Rph19*, *Rph21*, *Rph22*, *Rph24*, and *Rph25*) establish seedling resistance (Kavanagh et al., 2017). *Rph5* and *Rph6* on chromosome 3H (Zhong et al., 2003), *Rph9* and *Rph12* on chromosome 5H (Borovkova et al., 1998) and *Rph15* and *Rph16* on chromosome 2H have been described as alleles of the same gene (Weerasena et al., 2004). Only *Rph7*, *Rph15*, and *Rph16* are still effective in Europe (Niks et al., 2000; Perovic et al., 2004) and the number of effective *Rph* genes available to breeders is decreasing rapidly (Kavanagh et al., 2017). Among all known *Rph* genes, only *Rph1* has been isolated recently, using the newly developed cloning approach called Mutant Chromosome Sequencing (MutChromSeq) (Steuernagel et al., 2016) in combination with genetic mapping (Dracatos et al., 2018).

Molecular markers have been widely used in barley breeding for mapping of genes, marker-assisted selection, as well as in positional isolation of genes (Stein and Graner, 2005; Perovic et al., 2018). The most abundant molecular markers are single nucleotide polymorphism (SNP). Employing next generation sequencing (Ganal et al., 2018), SNPs are easily detectable in a high throughput manner and are therefore currently the markers of choice. The number of available SNP markers rapidly increased from about 180 EST markers to about 6,800 SNPs on the 9K Illumina iSelect chip up to 44,040 SNPs on the 50K Illumina Infinium array (Kota et al., 2003; Rostoks et al., 2005; Stein et al., 2007; Close et al., 2009; Muñoz-Amatriaín et al., 2011; Comadran et al., 2012; Bayer et al., 2017). The barley Genome Zipper (GZ) assembled 86% of the barley genes in a putative linear order (Mayer et al., 2011). Population sequencing methodology (POPSEQ) was developed as an integrated method to create a linear order of contigs using whole-genome-shotgun sequencing (WGS) data that resulted in the first ultra-high density map of the barley genome (Mayer et al., 2011; Mascher et al., 2013a). Assessment of the GZ and POPSEQ by Silvar et al. (2015) at seven loci mapped with higher genetic resolution revealed an accuracy of 97.8% with respect to the GZ and 99.3% to POPSEQ in comparison to consensus genetic maps. In addition to the above mentioned resources, advances in target capture/enrichment and next-generation sequencing, like GBS (Poland et al., 2012), exome capture (Mascher et al., 2013b), and barley reference genome sequence (Mascher et al., 2017) are available for marker development.

Although high resolution mapping allows precise zooming into targeted loci, the un-even distribution of crossovers along chromosomes (International Barley Genome Sequencing Consortium [IBSC], 2012) and the large variation in the genetic/physical ratio across the genome (Künzel et al., 2000) often hampers high-resolution genetic dissection. In barley, peri-centromeric regions (pCENR) comprise at least 48% of the physical genome but harbor only 14–22% of the total barley gene content (Mascher et al., 2017). The other extreme are hotspots of high recombination rates in telomeric regions (Bhakta et al., 2015). In case of the locus of *Ryd3*, which is located in a centromeric region, the physical/genetic ratio has been estimated at 14–60 Mb/cM, while the genome-wide average is 4.4 Mb/cM (Lüpken et al., 2014). At the *rym4/rym5* locus, the ratio of physical to genetic distances was in the range between 0.8 and 2.3 Mb cM and have increased to over 30 Mb cM, although the gene has been mapped on the telomeric region of chromosome 3H (Stein and Graner, 2005). This indicates that a large number of meiotic events is essential for a sufficient genetic resolution to detect recombination events in close vicinity to the targeted genes, and highlights the need for development of HRMPs.

Diverse collections of barley germplasm were evaluated for detecting new sources of leaf rust resistance (Perovic et al., 2003). In this respect, the *Rph*_*MBR*1012_ gene was mapped on the short arm of chromosome 1H (König et al., 2012), where only *Rph4* has previously been localized (McDaniel and Hathcock, 1969; König et al., 2012). Prior to the recently cloned gene *Rph1* by Dracatos et al. (2018), all efforts to isolate leaf rust resistance genes in barley were unsuccessful. An example of unsuccessful isolation is the case of *Rph*7 (Brunner et al., 2000; Scherrer et al., 2005). Hence, positional cloning is still one of the most efficient and reliable approaches to isolate a resistance gene in crop species with large genomes, such as wheat and barley (Krattinger et al., 2009). In barley, up to now five genes conferring resistance to fungal and viral pathogens have been isolated through map-based cloning, comprising *mlo* (Büschges et al., 1997; Simons et al., 1997), *Mla6* (Halterman et al., 2001), *Rpg1* (Brueggeman et al., 2002), *rym4/rym5* (Pellio et al., 2005) and *rym11* (Yang et al., 2014).

The aims of this study were to: (i) develop at HRMP for the *Rph*_*MBR*1012_ resistance gene, (ii) saturate the locus using all available state-of-the-art genomic resources i.e., GBS, 50K Infinium and the barley reference genome, (iii) anchor the genetic map to the barley reference sequence (iv) characterize the putative candidate rust resistance genes by allele specific re-sequencing and (v) test the developed markers for their diagnostic value.

## Materials and Methods

### Plant Material and Construction of a High-Resolution Mapping Population

For high resolution mapping of *Rph*_*MBR*1012_, a segregating population comprising of 4,775 F_2_ plants was constructed based on crosses between five DH-lines namely, the resistant (R) DH3/6 and DH3/127 and the susceptible (S) DH3/9, DH3/62 and DH3/74, which were derived from the original cross between the parental line MBR1012 (resistant) and Scarlett (susceptible). Based on these five DH-lines four crosses were conducted, namely DH3/74 (S) × DH3/6 (R), DH3/74 (S) × DH3/127 (R), DH3/6 (R) × DH3/9 (S) and DH3/62 (S) × DH3/127 (R) (Table 1). In order to identify recombinants, F_2_ plants were analyzed using two flanking co-dominant SSRs, i.e., QBS94 (distal) and QBS113 (proximal) (Perovic et al., 2013). Respective markers were analyzed by capillary electrophoresis at the genetic analyzer ABI PRISM^^®^^ 3100 (Applied Biosystems, Darmstadt, Germany). From identified heterozygous recombinant F_2_ plants in target interval, 12 progeny plants, representing F_3_ families were sown in 96 Quick pot plates. Genomic DNA of 10 days old plantlets was extracted in F_2_ and F_3_ according to Dorokhov and Klocke (1997). The quality of the extracted genomic DNA was checked by electrophoresis on 1% agarose gel and latter quantified by using the NanoDrop ND-100 spectrophotometer (PeQLab, Erlangen, Germany). By this approach, a HRMP of 537 recombinant inbred lines (RILs) was developed and subsequently used for marker saturation and resistance testing. Genomic DNA of the selected segmental homozygous RILs was extracted using the Miniprep method according to Stein et al. (2001). DNA of all samples was adjusted to a final concentration of 20 ng/μl. Furthermore, F_3_ recombinant plants were self-fertilized and as F_4_ segmental RILs used for phenotyping and genotyping with newly developed PCR based markers.

**Table 1:** DH lines and crosses used for the construction of the high resolution mapping population for *Rph_*MBR1012*_*.

Crosses	Number of analyzed F_2_	Number of selected segmental RILs (F_4_)		χ^2^ (*df* = 1, *p* < 0.05)
		Resistant	Susceptible	
DH3/74 (S) × DH3/6 (R)	389	32	29	0.1475
DH3/74 (S) × DH3/127 (R)	1469	88	72	1.6
DH3/6 (R) × DH3/9 (S)	713	45	53	0.653
DH3/62 (S) × DH3/127 (R)	2204	96	122	3.1009
Total	4775	261	276	0.4189

### Resistance Test

#### Inoculum Preparation

Fresh urediniospores of leaf rust isolate I-80 were prepared by artificial inoculation at the two-leaf stage of *Hordeum vulgare* cultivar Grossklappige, which is highly susceptible to the majority of *P. hordei* isolates. Inoculated plants were covered with plastic for 24 h at 18°C to ensure a moist environment. After 15 days, rust urediniospores were harvested and used for inoculation of RILs seedlings.

#### Resistance Tests

Resistance tests were carried out in the greenhouse by inoculation of RILs along with the two *H. vulgare* parental lines, i.e., MBR1012 (resistant), Scarlett (susceptible) and susceptible (DH3/62) and resistant (DH3/127) DH-lines as well as the cv. Grossklappige as a control. Three plants per segmental RILs were sown in 96 Quick pot trays and 10 days old plantlets were inoculated with fresh I-80 urediniospores according to Ivandic et al. (1998). Briefly, 10 mg of fresh spores were used per 100 plants and mixed with white clay (Laborchemie Apolda, Germany), (1:3). The inoculated plants were kept at 18°C and covered with plastic for 24 h, providing a moist environment for successful infection. All plants were scored at two time points, i.e., 10 and 13 days post-inoculation (dpi) according to Levine and Cherewick (1952). Segregation of resistant and susceptible plants was analyzed using the Chi-square (χ^2^) tests for goodness-of-fit to the expected Mendelian segregation ratios.

### Marker Development

For marker saturation, initially 6 Simple Sequence Repeats (SSRs), 7 size polymorphism and 24 SNPs markers derived from the barley GZ and 9K iSelect high-density custom genotyping bead chip were used for random saturation of the large interval of about 8 cM (Perovic et al., personal communication), while the Illumina 50K Infinium array and Genotyping By Sequencing (GBS) were used in combination with the barley reference sequence (Mascher et al., 2017) for very precise marker saturation within an interval of 0.1 cM of the locus in this study (Supplementary Table S1).

#### 50K iSelect Illumina SNP Array

The genomic DNA of parental lines, two DH-lines and two RILs from HRMP (carrying critical recombination within the resistance locus region) were used for the identification of polymorphic SNPs derived from the 50K Infinium array (TraitGenetics Gatersleben, Germany). The polymorphic SNPs located in the target interval were converted into Kompetitive Allele Specific PCR (KASP) assays by designing the two allele-specific forward primers, and one common reverse primer spanning the sequence of interest carrying the SNP position using Primer3 v. 0.4.0^1^ (Koressaar and Remm, 2007; Untergasser et al., 2012). KASP markers were then used for genotyping of the HRMP.

#### Genotyping-by-Sequencing (GBS)

The same lines as for the 50K array were used for GBS screening. A 20 ng/μl of genomic DNA of each line was used for GBS according to Wendler et al. (2014). Sequencing of selected lines was done on Illumina^^®^^ MiSeq^TM^ (Illumina, San Diego, United States). Sequencing data were analyzed using the Galaxy platform (Blankenberg et al., 2001; Giardine et al., 2005; Goecks et al., 2010) implemented at the JKI. After adapter and quality trimming (trim galore version 0.2.8.1; quality < 30, read length > 50), read mapping of the GBS data was executed using BWA version 0.7.15-r1140 (Li and Durbin, 2009) with standard settings to map the reads to the pseudomolecules of barley (Mascher et al., 2017). SNP calling was performed using mpileup version 1.2 (Li and Durbin, 2009), with genotype likelihood computation. Missing data was imputed with Beagle v4.1 (Browning and Browning, 2016). Biallelic SNPs were detected and subsequently filtered for differences between the resistant and susceptible parental lines and a minimum coverage of five reads per SNP using SnpSift version 4.2 (Cingolani et al., 2012). KASP markers were designed for polymorphic SNPs positioned in the target region^2^.

### Marker Saturation

The HRMP was genotyped using in total 56 molecular markers derived from the procedures described above. Molecular markers used may be divided in five types as follows: six SSRs based markers from the pyrosequencing assay (Silvar et al., 2011), three dominant present/absent markers, four size polymorphism markers [insertion/deletion polymorphisms (InDels)], 19 KASP markers and 24 Cleaved Amplified Polymorphic Sequences (CAPS) markers. Size polymorphisms markers and SSRs were amplified in a total volume of 10 μl, according to Perovic et al. (2013) and detected either using fluorescently labeled primers (M13) by capillary electrophoresis on the ABI Genetic Analyzer (ABI sequencer, ABI Perkin Elmer, Weiterstadt, Germany), or directly separated on a 1.5% agarose gel. For ABI analysis, 0.1 μl of M13 primer (10.0 pmol/μl)(5′-CACGACGTTGTAAAACGAC-3′) labeled with fluorescent dye was added to the reaction mix. One microliter of diluted PCR product was added to 14 μl of HiDi-Rox mastermix (1.4 ml Hidi and 6 μl Rox) in a total volume of 15 μl. Results were analyzed using the software package GeneMapper v4.0 (Applied Biosystems, Darmstadt, Germany). For 43 sequences, detected SNPs were converted either to KASP markers (see footnote 2) or CAPS markers using NEB cutter v.2.0^3^. KASP reaction was performed in total volume of 5 μl containing 2.5 μl KASP mix (LGC Genomics GmbH, Germany), 0.08 μl forward primer, allele 1 (10.0 pmol/μl, labeled with FAM M13 tail), 0.08 μl forward primer allele 2 (10.0 pmol/μl, labeled with HEX M13 tail), 0.2 μl reverse common primer (10.0 pmol/μl), and 2.2 μl template DNA (20 ng/μl). For CAPS analysis, DNA amplicons were cleaved with the respective restriction endonuclease (Table 2) in a volume of 20 μl, containing 2 μl corresponding 10× buffer, 0.1 μl appropriate enzyme, 7.9 μl HPLC gradient grade water (Carl Roth, Karlsruhe, Germany) and 8/10 μl of the PCR product. Proper temperature was applied according to manufacturer’s instructions for each restriction endonuclease and digestion was done for 3 h.

**Table 2:** Molecular markers used for the construction of the high resolution map.

Marker name	Marker type	Restriction enzyme	References
**(A) Genome Zipper**			
QBS70	CAPS	*Eco130I*	Rauser, 2012
QBS72	CAPS	*HpaII* (*MspI*)	Rauser, 2012
QBS73	CAPS	*BseN1* (*BsrI*)	Rauser, 2012
QBS74	CAPS	*HaeIII*	Rauser, 2012
QBS97	CAPS	*MlyI*	Perovic et al., 2012
QBS75	CAPS	*MfeI*	Rauser, 2012
QBS101	CAPS	*HhaI*	Perovic et al., 2012
QBS102	CAPS	*TaqI*	Perovic et al., 2012
QBS103	CAPS	*HpaII*	Perovic et al., 2012
QBS76	CAPS	*BamHI*	Rauser, 2012
QBS77	CAPS	*BfaI*	Rauser, 2012
QBS79	CAPS	*TaaI*	Rauser, 2012
QBS80	CAPS	*HpyCH4IV*	Rauser, 2012
QBS111	CAPS	*SspI*	Perovic et al., 2012
QBS112	CAPS	*BfaI*	Perovic et al., 2012
QBS100	CAPS	*HhaI* /*DdeI*	Perovic et al., 2012
QBS107	CAPS	*Eco47I* (*AvaII*)	Perovic et al., 2012
QBS71	Size polymorphism	–	Rauser, 2012
QBS98	Size polymorphism	–	Perovic et al., 2012
QBS99	Size polymorphism	–	Perovic et al., 2012
QBS106	Size polymorphism	–	Perovic et al., 2012
QBS78	±	–	Rauser, 2012
QBS110	±	–	Perovic et al., 2012
**(B) 9K iSelect**			
QBS105	CAPS	*Eco31I* (*BsaI*)	Perovic et al., 2012
QBS95	CAPS	*Hind III*	Perovic et al., 2012
QBS104	CAPS	*AciI* (*SsiI*)	Perovic et al., 2012
QBS108	CAPS	*HpyF10VI*	Perovic et al., 2012
QBS109	CAPS	*AjiI* (*BmgBI*)	Perovic et al., 2012
QBS94^1^	SSR	–	Perovic et al., 2012
QBS113^1^	SSR	–	Perovic et al., 2012
QBS96	±	–	Perovic et al., 2012
GBS 546	CAPS	*HhaI*	Kota et al., 2008
GBS 626	CAPS	*BtsCI*	Perovic et al., 2012
GBMS187	SSR	–	Li et al., 2003
GBS564	SSR	–	Perovic et al., 2012
QBS2	SSR	–	Stein et al., 2007; König et al., 2012
GBR534	SSR	–	Perovic et al., 2012

*^1^Initial flanking marker*

The following PCR conditions were used for all SSRs, size polymorphism and CAPS markers: denaturation at 94°C for 5 min followed by 12 cycles at 94°C for 30 s, annealing at 62°C to 56°C (–0.5°C/cycle) for 30 s, extension 30 s at 72°C, 94°C for 30 s, 56°C for 30 s, 72°C 30 s, 35 cycles, final extension at 72°C for 10 min.

The PCR amplification condition for KASP markers were: 10 min at 94°C, followed by 10 cycles: 94°C for 20 s, annealing at 61°C to 55°C (–0.6°C/cycle) for 60 s, followed by 26 cycles: 94°C for 20 s, 55°C for 60 s, 30°C 60 s. The real-time PCR machine was used to detect the fluorescence from HEX and FAM on plate reads. After thermal cycling was completed, the fluorescent signal was detected by reading the plate in the qPCR machine at 37°C. At the end of the run the results were shown in the data analysis software under “Allelic Discrimination.” The software automatically showed the clusters for the alleles for samples based on their position in the allelic discrimination plot (LGC, Guide to running KASP genotyping on the BIO-RAD CFX-series instruments’).

### Linkage Analysis

Linkage analysis was performed by dividing the number of the recombination events with the number of analyzed gametes, multiplied with 100. The recombination frequency was used for the genetic linkage map construction and visualized using MapChart (Voorrips, 2002) software package.

### Testing the Diagnostic Value of Co-segregating and Closely Linked Markers

Co-segregating markers in *Rph*_*MBR*1012_ locus were tested for their diagnostic value on a set of 63 genotypes comprising 25 selected barley genotypes/lines carrying *Rph1 to Rph25*, 23 parental lines and 15 Bowman introgression lines carrying *Rph1* to *Rph15* (Table 3). The diagnostic value of tested co-segregating markers (%) was calculated using the following equation:

$$\mathrm{Diagnostic value}=\frac{\mathrm{Number of lines showing different allele of MBR}1012}{\mathrm{Total number of analyzed lines}}\times 100(\%)$$

### Anchoring the Genetic Map to the Barley Reference Sequence

All 56 markers used for construction of the HRMP were anchored to the barley Reference genome sequence (Mascher et al., 2017). All sequences including forward and revers primers were blasted against the barley reference genome sequence^4^ using BLASTN algorithm applying default parameters. Obtained physical positions of mapped markers were visualized using software MapChart (Voorrips, 2002).

### Use of the Barley Reference Sequence for the Identification of Candidate Genes

Marker positions in the barley reference sequence were used to determine the target interval of the resistance gene locus and to extract putative candidate genes^5^. After defining the genomic region of the resistance locus at the barley reference sequence, High-Confidence (HC) and Low-Confidence (LC) genes including Exon-intron boundaries were extracted from the available annotation (Mascher et al., 2017). The reconstruction of the gene intron-exon-structure was performed using the internet platform “Splign” ^6^ from NCBI, which allows alignment of mRNA to genomic sequence (Kapustin et al., 2008).

### Allele Specific Re-sequencing of Candidate Genes

The allele specific re-sequencing of candidate genes was conducted for 18 high and 11 low confidence genes positioned in the candidate interval. Online software Primer3 v. 0.4.0 (see footnote 1) (Koressaar and Remm, 2007; Untergasser et al., 2012) setting the parameters at 20–22 bp, temperature 58–62°C and product size of 350 bp was used for primer design, which subsequently were then tested for their specificity for chromosome 1H using the barley blast server(see footnote 4) against the barley pseudomolecules according to Mascher et al. (2017). In the first round of low pass resequencing, a set of 36 primer pairs were designed covering all 29 high and low confidence genes. In the second round of the experiment, 25 primer pairs were designed in order to sequence the full length of five disease resistance genes. To sequence the entire gene, Morex contigs including the gene sequence of each disease resistance gene were identified using (see footnote 4) allowing to design primers at least 20 bases upstream of the start codon and 20 bases downstream of the stop codon. Moreover, the primers should overlap to ensure that there are no gaps between the fragments after sequence analysis. A fragment size of 400 to 1,200 bp was chosen because of the maximum sequencing length. Amplification was done on the parental genotypes MBR1012 and Scarlett, as well as on two DH-lines [DH3/62 (S), DH3/127 (R)]. Amplification reaction was prepared in a total volume of 20 μl containing 2 μl of 10× PCR buffer (Qiagen, Hilden, Germany), 2 μl of 25 mM MgCl2, 0.4 μl of 10 mM dNTPs (Fermentas, Schwerte, Germany), 0.5 μl of each forward and reverse primer (10.0 pmol/μl), 0.16 unit of fire DNA polymerase (5 U/μl), (Qiagen, Hilden, Germany), 12.44 μl HPLC gradient grade water (Carl Roth, Karlsruhe, Germany) and 2 μl of template DNA (20 ng/μl). Next, obtained PCR products of the same size were subjected for sequencing. PCR fragments were separated by agarose gel electrophoresis and analyzed using the imaging system Gel Doce^TM^ XR and the Quantity One^^®^^ 1-D analysis software (4.6.2) (Bio-Rad, Hercules, United States) and subsequently sequenced by the company Microsynth AG (Balgach, Switzerland) using the Sanger sequencing method (Sanger et al., 1977). Obtained sequences were edited and analyzed using Sequencher 5.1 software (Gene Codes, Ann Arbor, MI, United States) using default parameters.

Functional analysis of identified polymorphisms between parental lines (MBR1012 and Scarlett) was done using the multiple sequence alignment program, MAFFT by default parameters (Katoh and Standley, 2013).

**Table 3:** Selected Bowman lines and parental lines carrying 25 known Rph genes for diagnostic value evaluation of developed markers linked to the resistance locus, size of alleles and restriction patterns.

	Cultivar/lines	*Rph-Gen*	Gen Locus	QBS128	QBS116	QBS117	GBS626	GBR534	GBS546	Locus
1	**MBR1012**	Resistance/*Rph*_*MBR*1012_	1HS	T	C	C	300–400	358	490	König et al., 2012
2	**Scarlett**	*Rph3*/*Rph9*/*Rph12*	7HL/5HS/5HL	C	T	T	400	Null	330	Jin et al., 1993; Borovkova et al., 1998
3	**Oderbruker**	*Rph1*	2H	C	C	C	400	358	330	Tuleen and McDaniel, 1971; Tan, 1978
4	**B.L.195-246-1**	*Rph1*	2H	C	C	C	400	358	330	Roane and Starling, 1967
5	**Peruvian**	*Rph2*	5HS	T	C	C	300–400	358	490	Franckowiak et al., 1997; Borovkova et al., 1997
6	**B.L.195-266-1**	*Rph2*	5HS	C	T	C	400	358	330	Borovkova et al., 1997
7	**B.L.193-343-1**	*Rph2*	5HS	T	C	C	400	358	330	Borovkova et al., 1997
8	**Estate**	*Rph3*	7HL	T	C	C	300–400	358	330–490	Jin et al., 1993
9	**B.L.195-267-2**	*Rph3*	7HL	C	T	C	400	358	330	Jin et al., 1993
10	**Gold**	*Rph4*	1HS	C	C	C	400	358	490	McDaniel and Hathcock, 1969
11	**B.L.195-268-4**	*Rph4*	1HS	C	C	C	400	358	490	McDaniel and Hathcock, 1969
12	Magnif	*Rph5*	3HS	T	C	C	300–400	358	490	Mammadov et al., 2003
13	**B.L.195-269-1**	*Rph5*	3HS	T	C	C	300–400	352–358	490	Mammadov et al., 2003
14	**Bolivia**	*Rph2+6*	5HS+3HS	T	C	C	300–400	358	490	Zhong et al., 2003
15	**B.L.195-270-2**	*Rph6*	3HS	C	T	C	400	358	330	Brunner et al., 2000
16	**Cebad capa**	*Rph7*	3HS	T	C	C	400	358	330	Brunner et al., 2000; Graner et al., 2000
17	**B.L.193-21**	*Rph7*	3HS	C	T	C	400	358	330	Brunner et al., 2000
18	**B.L.196-424-1**	*Rph7*	3HS	C	C	H	400	338–358	330	Brunner et al., 2000
19	**Egypt4**	*Rph8*	7HS	C	C	C	400	358	330	Borovkova et al., 1997
20	**B.L.195-349-4**	*Rph8*	7HS	C	C	C	400	358	330	Borovkova et al., 1997
21	**Trumph**	*Rph9+12*	5HL	C	C	C	400	358	490	Borovkova et al., 1998
22	**B.L.194-224**	*Rph9*	5HS	C	T	C	400	358	330	Borovkova et al., 1998
23	**B.L.195-274-1**	*Rph9*	5HS	C	T	C	400	358	330	Borovkova et al., 1998
24	BC8	*Rph10*	3HL	T	C	C	300–400	358	330–490	Feuerstein et al., 1990
25	**B.L.195-272-1**	*Rph10*	3HL	C	T	C	400	358	330	Feuerstein et al., 1990
26	BC67	*Rph11*	6HS	T	C	C	300–400	358	330–490	Feuerstein et al., 1990
27	**B.L.195-273-2**	*Rph11*	6HS	C	T	C	400	358	330	Feuerstein et al., 1990
28	**B.L.195-288-2**	*Rph13*	7HS	C	T	C	400	358	330	Sun and Neate, 2007
29	**B.L.195-290-2**	*Rph14*	7HS	C	T	C	400	358	330	Golegaonkar et al., 2009
30	**B.L.195-282-2**	*Rph15*	2HS	C	T	C	400	358	330	Weerasena et al., 2004
31	**Hordeum spontaneum 680**	*Rph16*	2HS	C	–	C	400	358	330	Ivandic et al., 1998
32	NGB22914	*Rph17*	2HS	C	C	C	400	358	490	Pickering et al., 1998
33	NGB22900	*Rph18*	2HL	C	C	C	400	358	330–490	Pickering et al., 2000
34	Prior	*Rph19*	7HL	T	C	C	300–400	358	330–490	Park and Karakousis, 2002
35	Flagship	*Rph20*	6H	C	C	C	400	358	330–490	Hickey et al. (2011)
36	Ricardo	*Rph21*	4H	T	C	C	400	358	330	Sandhu et al., 2012
37	NGB22893	*Rph22*	2HL	T	C	C	400	358	330	Johnston et al., 2013
38	Yerong	*Rph23*	7HS	T	C	T	400	Null	330	Singh et al., 2015
39	ND24260-1	*Rph24*	5HS	T	C	T	400	Null	330	Ziems et al., 2017
40	Fongtien	*Rph25*	5HL	C	C	C	400	358	330	Kavanagh et al., 2017
41	**Reka1**	*Rph3+?*	7HL+?	T	C	C	300–400	358	490	Jin et al., 1993
42	**HOR4280**	*Rph1d+1r*	2H	C	T	C	400	358	330	Roane and Starling, 1967
43	**Bowman**	*Susceptible*	–	C	T	C	400	358	330	–
44	**Bowman**	*Rph15*	2HS	C	T	C	400	349–358	330	Weerasena et al., 2004
45	**HOR500-1**	*Rph1d+1r*	2H	C	–	C	400	358	490	Roane and Starling, 1967
46	**Grossklappige**	*Susceptible*	–	T	C	H	400	338–358	330	–
47	**Sudan**	*Rph1*	2H	C	C	C	400	351–358	330	Roane and Starling, 1967
48	**Quinn**	*Rph2+5*	5HS+3HS	T	C	T	400	338–358	330	Borovkova et al., 1997; Mammadov et al., 2003
49	**Rika × F1**	*Rph3*	7HL	T	C	C	400	352–358	330	Jin et al., 1993
50	**Lada**	*Susceptible*	–	C	T	C	400	358	490	–
51	**Krona**	*Rph12*	5HL	C	C	C	400	358	330	Borovkova et al., 1998
52	**Alexis**	*Susceptible*	–	C	T	C	400	358	330	–
53	**HOR679-3**	*Rph3*	7HL	C	C	C	400	352–358	330	Jin et al., 1993
54	**Vada**	*Partial res.*	–	C	C	C	400	352–358	490	–
55	**HOR1132**	*Rph2r*	5HS	T	C	C	300–400	352	330–490	Borovkova et al., 1997
56	**HOR1063**	*Partial res.*	–	C	T	C	400	358	330–490	–
57	**Salome**	*Susceptible*	–	C	T	C	400	358	330	–
58	**HOR2596**	*Rph9*	5HS	C	C	C	400	358	330	Borovkova et al., 1998
59	**Emir**	*Susceptible*	–	C	C	C	400	358	330–490	–
60	**Karat**	*Susceptible*	–	T	C	C	400	352–358	330	–
61	**L94**	*Susceptible*	–	C	T	C	400	349–358	330	–
62	**MBR532**	*Susceptible*	–	C	C	C	400	358	330	–
63	**Igri**	*Susceptible*	–	C	H	T	400	338–358	330	–
**Diagnostic value (%)**				**67.21**	**36.06**	**9.8**	**83.6**	**24.59**	**80.32**	

*Bold fonts indicate the selected 51 lines from König et al. (2012) and underlined ones are 12 new lines analyzed.*

## Results

### Construction of the High-Resolution Mapping Population

Four crosses i.e., DH 3/74 (S) × DH3/6 (R), DH3/74 (S) × DH3/127 (R), DH3/6 (R) × DH3/9 (S) and DH3/62 (S) × DH3/127 (R), were used for the construction of the HRMP (Table 1). In total, of 5,237 F_2_ plants, 4,775 survived, and from corresponding F_3_ families 537 recombinant F_4_ RILs were developed, resulting in an interval harboring the resistance locus of 0.07% recombination. Finally, a genetic resolution of 0.010% recombination was achieved.

**FIGURE 1:**
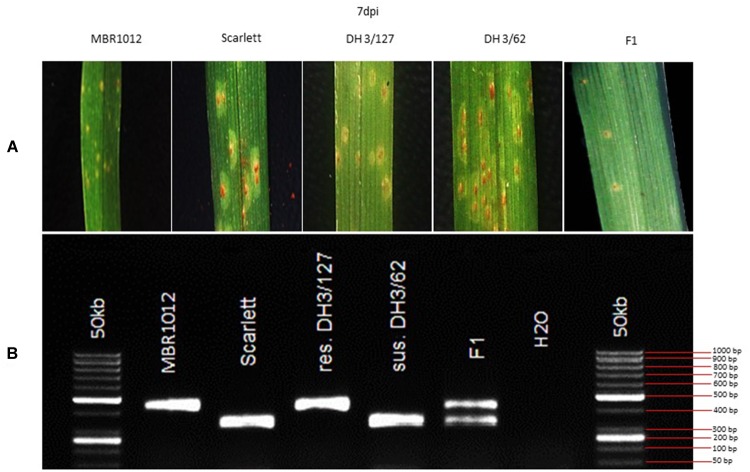
**(A)** Macroscopic symptoms of *Puccinia hordei* isolate I-80 on the resistant parent (MBR1012), the susceptible parent (Scarlett), two DH lines and the F_1_ 7 days post-inoculation. **(B)** CAPS marker GBS546 originated from a high-confidence gene in the target interval.

Phenotypic analysis of resistance to *Rph*_*MBR*1012_ showed a segregation of 261 resistant and 276 susceptible RILs and revealed the expected 1r:1s segregation ratio among these RILs. Chi-square test (χ^2^ 1:1 = 0.4189, *df* = 1, *p* < 0.05) for goodness of fit indicated that the resistance in MBR1012 is monogenically controlled (Figure 1 and Table 1).

### Marker Saturation of the *Rph*_*MBR*1012_ Locus and Anchoring to the Barley Reference Sequence

**FIGURE 2:**
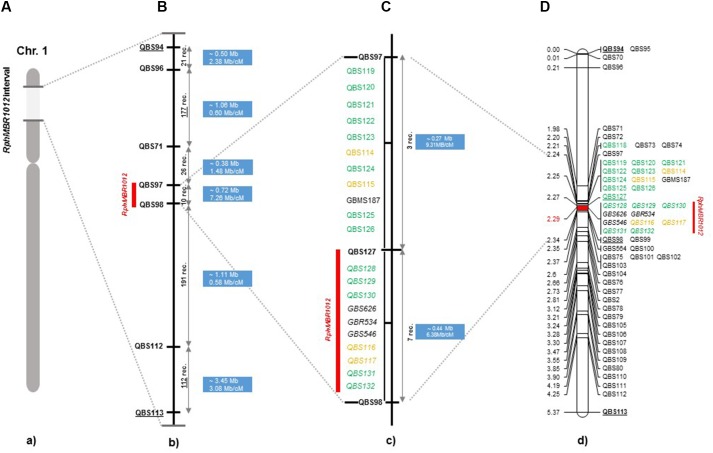
High-resolution genetic map of *Rph*_*MBR*1012_. **(A)** The genomic region harboring the *Rph_MBR1012._* **(B)** The identification of 537 recombinants and mapping of *Rph*_*MBR*1012_ locus based on markers derived from the Genome Zipper and the 9K iSelect chip flanked by QBS97 and QBS98. The blue boxes indicate the physical size. **(C)** Target region used for marker saturation based on the 50K Infinium chip and GBS markers. Markers derived from the 50K Infinium chip are highlighted with orange and markers derived from GBS are shown in green. Co-segregating markers are indicated by italics. **(D)** Genetic map of *Rph*_*MBR*1012_ locus.

A fine map of the *Rph*_*MBR*1012_ was constructed using the set of 537 segmental homozygous RILs (Figure 2). Marker saturation of the HRMP resulted in reducing the target interval to 0.1 cM. After screening parental lines using the 50K chip and GBS, 19 new polymorphisms were identified in the target region of 0.1 cM.

**FIGURE 3:**
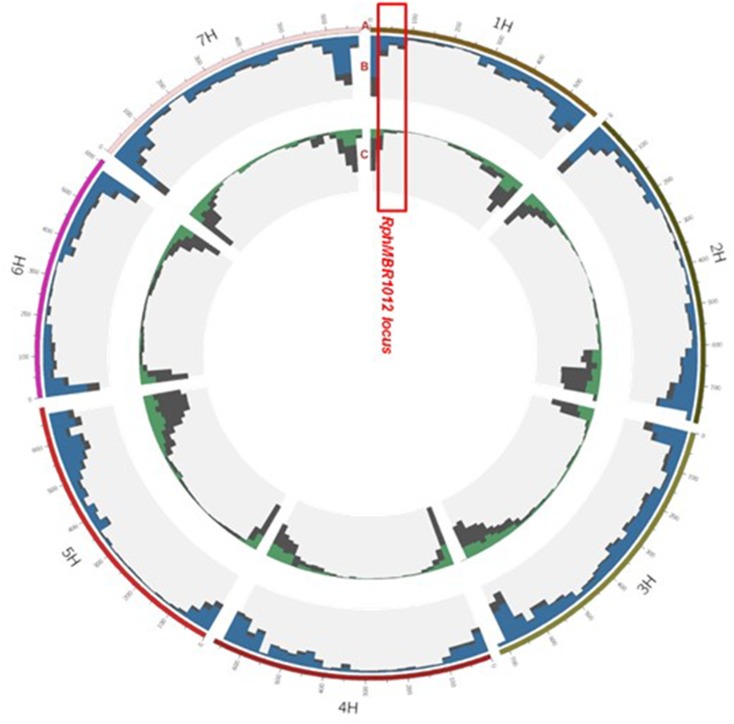
Landscape of the 50K and GBS marker distribution. Track A: gives the seven barley chromosomes. Track B: grey color depicts GBS: (all, 48.226), blue, position of 37,287 polymorphic SNPs between MBR1012 and Scarlett. Track C: distribution of SNP Chip (50K) markers: grey (all, 40,777), green, position of 14,616 polymorphic SNPs between MBR1012 and Scarlett.

The 50K screen revealed in total, a set of 40,777 scoreable SNPs at the barley genome (Figure 3). Out of these, 14,616 SNPs showed homozygous polymorphisms between resistant and susceptible genotypes. Thirty-nine SNPs were located at the large interval of 8.0 cM on chromosome 1HS, and four SNPs were located at the closest target interval comprising 0.1 cM. These SNPs were converted into KASP markers and mapped on the whole HRMP population (Supplementary Table S1).

Genotyping by sequencing analysis yielded 48,226 SNPs distributed over all seven barley chromosome, of which 37,287 showed homozygous polymorphisms between resistant and susceptible lines (Figure 3). Out of these, 80 polymorphic markers were located in the larger interval, flanked by QBS94 and QBS113 (8.0 cM) and 15 SNPs were identified in the shortened interval of 0.1 cM. KASP markers were designed for all 15 SNPs and used for genotyping of the 537 RILs (Supplementary Table S1).

**FIGURE 4:**
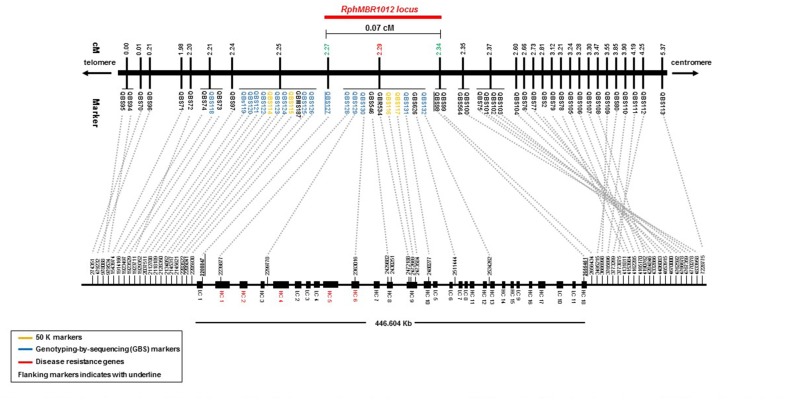
High-density genetic and physical map of the *Rph*_*MBR*1012_ region on barley chromosome 1HS based on 56 molecular markers and 537 recombinant inbred lines derived from the cross MBR1012 × Scarlett.

**FIGURE 5:**
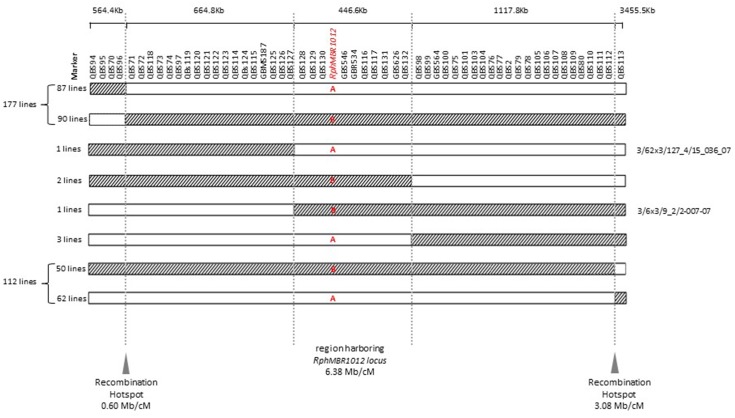
Graphical genotypes of F_4_ RILs for all 537 recombinant lines carrying cross-over events between QBS94 and QBS113 (8.0 cM). A – (Susceptible genotype = white) and B – (Resistant genotype = hatched) in the target locus indicate the result of the resistance test of recombinant lines. Border of hatched to white shows the recombination position between the MBR1012 allele to the Scarlett allele and white to hatched shows the recombination position between the Scarlett allele to the MBR1012 allele.

Mapping of all mentioned markers showed that the *Rph*_*MBR*1012_ locus is located in a region of 0.07 cM between tightly linked markers QBS127 (SNP) and QBS98 (size polymorphism) at 0.020% (distal) and 0.050% (proximal) recombination of the *Rph*_*MBR*1012_ locus. Thus, the target interval was shortened from 0.1% recombination to 0.07% recombination (Figure 4). A high-density genetic map revealed ten markers co-segregating (QBS128, QBS129, QBS130, QBS131, QBS132, GBS626, GBR534, GBS546, QBS116 and QBS117) within the *Rph*_*MBR*1012_ locus (Figure 4). Moreover, recombination distribution in the target interval was uneven varying from 0.58 to 0.60 Mb/cM proximally and distally, respectively to the resistance locus, to 7.26 Mb/cM at the *Rph*_*MBR*1012_ locus (Figure 2). Marker saturation also revealed a high number of recombination between markers QBS96 and QBS71 in the distal region of the interval, i.e., 177 recombination events and 112 recombinations between markers QBS112 and QBS113 located proximally (Figure 5). However, the analysis allowed narrowing the *Rph*_*MBR*1012_ locus to a region comprising a limited number of candidate genes.

BLAST searches against the barley reference sequence revealed that the mapped markers were in a nearly perfect co-linear order. However, 15 markers within 1.34 Mb in the distal part of chromosome 1HS showed a marker inversion (Figure 4). The BLAST searches also indicated only one hit for 20 markers (12 SNPs, 4 SSRs and 4 size polymorphism) on chromosome 1H and two or more hits for 17 markers (12 SNPs, 2 SSRs and 3 size polymorphism). The physical size of the large target interval of 8.0 cM between the flanking markers QBS94 and QBS113 encompassed 6.24 Mb. This region harbors 299 genes of which 183 are high confidence (HC) genes and 116 are low confidence genes. Based on the sequence annotation of HC and LC genes, 23 genes were disease resistance proteins and three were annotated as powdery mildew resistance proteins (Supplementary Table S2). Likewise, physical size of the shortened interval carrying *Rph*_*MBR*1012_ flanked between QBS127 and QBS98 was estimated to 0.44 Mb (Figure 4). In this interval 11 low confidence and 18 high confidence (HC) genes were detected (Supplementary Table S2). Fifteen of these genes are functionally annotated and five of them are related to pathogen resistance, i.e., HORVU1Hr1G000830 (disease resistance protein), HORVU1Hr1G000840 (powdery mildew resistance protein PM3 variant), HORVU1Hr1G000860 (disease resistance protein), HORVU1Hr1G000900 (disease resistance protein) and HORVU1Hr1G000910 (disease resistance protein) (Supplementary Table S2). The markers QBS128 and QBS130 are exactly located at two disease resistance genes, namely HORVU1Hr1G000830 and HORVU1Hr1G000910.

**FIGURE 6:**
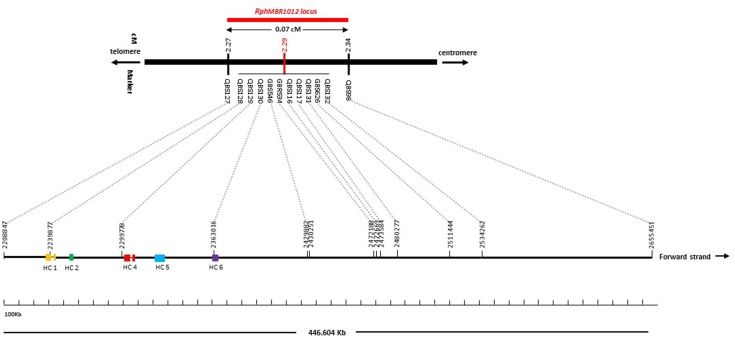
Gene structure of five disease resistance genes positioned in target interval. Colored boxes in genes indicate CDSs (exon) and thin lines indicate introns.

Furthermore, the available barley annotation (Mascher et al., 2017) revealed a mosaic structure of exon and intron fragments only for two disease resistance genes, namely HORVU1Hr1G000830 and HORVU1Hr1G000860, while the three other disease resistance genes (HORVU1Hr1G000840, HORVU1Hr1G000900 and HORVU1Hr1G000910) only have one coding exon (Figure 6).

### Testing Diagnostic Value of Developed Markers

Diagnostic assessment of markers co-segregating markers with the *Rph*_*MBR*1012_ was conducted. However, out of ten tested markers only six showed clear a allele differentiation, whereas for four markers, i.e., QBS129, QBS130, QBS131 and QBS132 had to be excluded. The number of alleles detected varied from two alleles for markers QBS116, QBS117, QBS128, QBS130, GBS546, GBS626 and seven alleles for GBR534. For the two markers GBS546 and GBS626 most of the cultivars/lines showed the same allele as the susceptible parental line Scarlett with 80.32 and 83.60% accuracy, respectively. Marker QBS117 with 9.8% accuracy for *Rph*_*MBR*1012_ has no diagnostic value to trigger this gene. Other tested markers were also of limited value for marker-assisted selection (Table 3).

### Allele Specific Re-sequencing of Candidate Genes

Allele specific re-sequencing for all 29 putative genes located on the pseudomolecule of chromosome 1H from 2,206,515 to 2,763,382 bp located in a narrowed interval comprising 0.44 Mb was conducted twice. In the first round of low pass resequencing, a set of 36 primer pairs were designed, 33 primer pairs amplified products in both parental lines, one was dominant by amplifying products in Scarlett and two were dominant for MBR1012 and did not produce any fragment on Scarlett. For two genes no specific primer on chromosome 1HS could be designed due to the high similarity of the sequences of these genes (e.g., gene HORVU1Hr1G000820.1: on chromosome 4H, 1863 bp of 1866 bp identical to chromosome 1H). Out of 36 primer pairs, 24 primer pairs were functional, while 12 primer pairs were not functional, since PCR products gave multiple bands, smear or present/absent patterns. Finally, 24 PCR amplicons of the functional primer pairs were sequenced. Moreover, markers for which polymorphisms were based on size polymorphism of polymerase chain reaction (PCR) fragments between parental lines (HORVU1Hr1G000910.9_s3958_as4143 and HORVU1Hr1G001060.1_s173_as480) were directly mapped into the HRMP population. By editing the sequence data, sequence of 18 amplicons could be aligned in both parental lines while for six fragments no alignments were achieved due to the low quality of the sequence data or obtained heterozygous signals (Supplementary Table S3).

Next, for whole length amplification and re-sequencing of five disease resistance genes in the target interval, 25 new primer pairs were designed (Supplementary Table S4). Out of 25 designed primers, 23 amplified products in both parental lines. From this experiment, 12 PCR products were sequenced (Supplementary Table S3). Finally, for 31,204 bp of all 29 candidate genes 61 primer pairs were designed, yielding DNA sequence information for 17,107 bp in MBR1012 and 16,963 bp in Scarlett. Using this sequence data, 259 SNPs were identified for disease resistance genes from the target interval. Moreover, from gene HORVU1Hr1G000900.5 (Disease resistance protein) a large deletion (InDel) was identified in Scarlett ranging from 26 to 222 bp. Seven SNPs for HORVU1Hr1G000830.3, nine for HORVU1Hr1G000860.7 and 243 for HORVU1Hr1G000900.5 were identified (Supplementary Table S3). For two resistance genes i.e., HORVU1Hr1G000840.1 and HORVU1Hr1G000910.9 no SNP/InDel were identified. Functional annotation of defined SNPs between parental lines, MBR1012 and Scarlett, revealed synonymous mutations for 11 SNPs whereas for 17 SNPs amino acid substitutions were detected. For two SNPs the arginine amino acid changed to a stop codon (TGA) (Table 4). Multiple alignment also revealed polymorphisms between the parents and barley reference sequence (Supplementary Table S5).

## Discussion

Leaf rust is an important fungal disease affecting barley production (Park, 2003). Fungicide application is an option to reduce yield losses but is not always efficient and cannot be considered as a sustainable disease management (Park et al., 2015). Thus, growing of resistant cultivars is the most economical and environmental friendly way to reduce yield losses caused by leaf rust (Kolmer, 1996). However, disease resistance provided by major *Rph* genes is often overcome due to the emergence of new *P. hordei* pathotypes (Niks, 1982; Steffenson et al., 1993; Park, 2003) indicating the need for introducing new sources of resistance into barley breeding as well as the need for isolating known ones toward deciphering the structure and function offering the possibility of developing functional markers for breeding and create new alleles by e.g., CRISPR/Cas9 (Kumar et al., 2018).

In this study we have shown the efficient use of the barley reference sequence in physical mapping and especially in marker saturation. Previously, Perovic et al. (2003) demonstrated that the barley landrace MBR1012 is resistant to the barley leaf rust isolate I-80, which later was mapped using 14 SSRs and three SNPs markers on barley chromosome 1HS (König et al., 2012). A null allele of the SSR marker GBMS187 was identified as the closest linked marker at 0.8 cM proximal to the resistance gene. The allelic status of *Rph*_*MBR*1012_ and *Rph4* (McDaniel and Hathcock, 1969), two genes mapped on the short arm on barley chromosome 1HS, is part of an ongoing experiment (Perovic et al., in preparation). The phenotypic evaluation conducted here revealed a hypersensitive reaction of the *Rph*_*MBR*1012_ resistance gene (Figure 1), while the genetic analysis demonstrates that by using genetically mapped markers in combination with the genome sequence information (Mascher et al., 2017) the physical position of this locus can be determined easily. An initial size of the locus of 6.25 Mb that was estimated based on the published map was further downsized by the use of new marker resources and by increasing the genetic resolution.

**Table 4:** Functional annotation of SNPs between parental lines (MBR1012 and Scarlett) originated from candidate genes located within the 0.44 Mb of target interval.

Gene	Alignment position	Type of mutation	Codon	Amino acid substitution	Mutation/SNP			
					Cultivar: MBR1012		Cultivar: Scarlett	
					Position	Nucleotide	Position	Nucleotide
**LC**								
HORVU1Hr1G000880	197	E	TTG -> TTT	L -> F	122	A	122	C
	250	E	CGA -> TGA	R -> ^∗^	175	A	175	G
HORVU1Hr1G000970	482	E	AGA -> TGA	R -> ^∗^	187	T	187	A
HORVU1Hr1G001100	1779	I			219	A	219	G
	1780	I			220	T	220	A
**HC**								
HORVU1Hr1G000830	3405	E	GCA -> GCC	synonymous	39	T	39	G
	3573	E	GAT -> GAC	synonymous	207	G	207	A
	3782	E	AGT -> CGT	S -> R	78	G	78	T
	4521	E	TTT -> TTC	synonymous	231	A	231	G
	4736	E	GAG -> AAG	E -> K	446	C	446	T
	4854	U			564	G	564	C
	4887	U			597	A	597	G
HORVU1Hr1G000860	2639	I			153	T	153	C
	2651	I			165	A	165	G
	2799	E	CAG -> CAA	synonymous	308	C	308	T
	2834	E	TCT -> GCT	S -> A	343	C	343	A
	2908	E	AGT -> ATT	S -> I	417	A	417	C
	2917	E	CGA -> CAA	R -> Q	426	C	426	T
	2967	E	CTG -> CTT	synonymous	476	A	476	C
	3534	E	CTC -> CTT	synonymous	79	A	79	G
	3785	E	ACA -> GCA	T -> A	330	T	330	C
HORVU1Hr1G000920	1091	E	CCC -> CCT	synonymous	99	G	99	A
	1112	E	ACT -> ACA	synonymous	120	A	120	T
	1185	E	GGC -> GCC	G -> A	193	G	193	C
HORVU1Hr1G000930	171	E	CCG -> TCG	P -> S	44	A	44	G
	207	E	CTG -> GTG	L -> V	80	C	80	G
	218	E	GTT -> GCT	V -> A	91	G	91	A
	230	E	CTT -> CAT	L -> H	103	A	103	T
	245	E	CAC -> CTC	H -> L	118	A	118	T
	266	E	CAG -> CGG	Q -> R	139	T	139	C
	271	E	GTG -> GTA	synonymous	144	T	144	C
HORVU1Hr1G000960	930	E	CGG -> CAG	R -> Q	260	C	260	T
	1071	I			401	G	401	A
	1082	I			412	G	412	T
	1337	I			156	T	156	A
	1341	I			160	A	160	G
	1379	I			198	A	198	G
HORVU1Hr1G001040	90	E	GAC -> GAT	synonymous	22	T	22	A
	100	E	AAC -> ATC	N -> I	32	G	32	A
	213	E	GCC -> GCT	synonymous	145	A	145	G
HORVU1Hr1G001060	615	E	GGA -> CGA	G -> R	112	G	112	C
	635	I			132	T	132	A
	651	I			148	G	148	A
	714	I			211	T	211	C

*^∗^: Stop codon; E: Exonic; I: Intronic; U: Upstream; D: Downstream.*

For many years, mapping of resistance genes relied on the use of various molecular markers i.e., restriction fragment length polymorphism (RFLP) (Graner et al., 1991; Kleinhofs et al., 1993), random amplified polymorphic DNAs (RAPDs) (Williams et al., 1990; Chalmers et al., 1993), amplified fragment length polymorphism (AFLPs) (Vos et al., 1995; Qi et al., 1998) and SSRs (Ramsay et al., 2000; Varshney et al., 2007). For instance, the powdery mildew resistance gene *mlo* was identified by a combined use of RFLP and AFLP markers which was the first gene, isolated by map-based cloning in barley (Büschges et al., 1997). AFLP, RAPD and RFLP-derived markers were also used to saturate the *Mla* region (Wei et al., 1999). However, using these marker systems, gene isolation was a laborious and time consuming effort. Advances in molecular marker technologies as well as the previous version of the barley genome sequence already facilitated an accelerated fine mapping of disease resistance genes (Lüpken et al., 2013, 2014; Yang et al., 2014). New Illumina SNP genotyping assays, namely 9K and 50K (Comadran et al., 2012; Bayer et al., 2017), together with GBS (Poland et al., 2012) opened a new way for a more efficient and faster marker saturation of target loci in barley. In our study, above mentioned marker resources were used for a first marker saturation of *Rph*_*MBR*1012_. During the simultaneous construction of a fine map and an initial marker saturation a set of 37 GZ and 9K iSelect SNP markers were randomly selected and mapped to our target interval of 8.0 cM reducing the target interval to 0.1 cM flanked by QBS97 and QBS98. Subsequently, the newly developed high-density barley 50K Infinium SNP markers (Bayer et al., 2017) and GBS markers, which were selected using the reference sequence in the shortened candidate interval (0.1 cM), resulted in the identification of nineteen additional polymorphic SNPs. These markers were converted into KASP markers and the *Rph*_*MBR*1012_ locus was genetically further narrowed into an interval of 0.07 cM between the markers QBS127 and QBS98. In the target interval, ten markers i.e., QBS128, QBS129, QBS130, GBS626, GBR534, GBS546, QBS116, QBS117, QBS131 and QBS132 spanning 0.07 cM genetic distance between QBS127 (at 0.02 cM) and QB98 (at 0.05 cM) were co-segregating. Seven out of the ten co-segregating markers, namely QBS116 (50K), QBS117 (50K), QBS128 (GBS), QBS130 (GBS), QBS131 (GBS), GBS546 and GBR534, were located in five genes in the target interval.

Fine mapping of resistance genes is a bottleneck in gene isolation due to the presence of many genes within target intervals, an uneven recombination frequency and a lack of molecular markers, (Stein and Graner, 2005). The fine map for the *Rph*_*MBR*1012_ region constructed in this study was based on a set of 56 molecular markers including four InDel, three present/absent, six SSRs, and 43 SNPs markers. Even though *Rph*_*MBR*1012_ is located in the telomeric region, it turned out that recombination events are not distributed continuously along this region. Although *Rph*_*MBR*1012_ is surrounded with two highly recombining regions at the telomere of chromosome 1HS, 0.58 and 0.6 Mb/cM, the locus is in very unfavorable region of 7.28 Mb/cM with a high number of co-segregating markers, again elucidating that the potential of map-based cloning still depends on the genomic context around the gene of interest. Uneven distribution of recombination frequencies along the genome (Künzel et al., 2000; Akhunov et al., 2003) and differences in local recombination rates, may cause regions even without any recombination over large physical distances which are not suited to map based cloning (Qi and Gill, 2001; Neu et al., 2002). Consequently, the efficiency of an effort of increasing the population size has always to be considered.

Genome-wide studies and multiple gene surveys recorded variation of the SNP frequencies in barley from one SNP per 240 bp, per 200 bp, and per 189 bp (Shavrukov, 2016). In contrast, one SNP per 7 bp in the leaf rust resistance *Rph7* gene region evidently showed the usefulness of high-density SNP markers for the purpose of gene isolation in barley (Scherrer et al., 2005).

In addition to the resources used in our study the following genomic resources for marker saturation nowadays may be used: exome sequencing (Mascher et al., 2013b), RNA sequencing (RNAseq) (Wang et al., 2009) which is based on transcriptome profiling, resistance gene enrichment sequencing (RenSeq) (Andolfo et al., 2014) and WGS. These methods may serve to enhance the detection of polymorphism in the genome and to develop markers toward gene isolation in a short period of time. More recently, MutRenSeq that combines the complexity reduction of R gene targeted enrichment sequencing and computational analysis based on comparative genomics provides a tool for the rapid cloning of disease resistance (R) genes in plants (Steuernagel et al., 2016; Dracatos et al., 2018).

Anchoring of the markers against the barley reference sequence elucidated the physical size of 0.44 Mb for the interval harboring *Rph*_*MBR*1012_. The order of all mapped markers were inconsistent with the order in the barley physical map (Stein et al., 2007). However, a large rearrangement of 15 markers within 1.34 Mb in the distal part of chromosome 1H was observed. This inversion is based on non-fixed orientation of the BAC-based sequence contig within the small scaffold having only one the anchor point (personal communication Martin Mascher).

Twenty-nine annotated genes were identified within the narrowed down interval between markers QBS127 and QBS98 comprising five disease associated resistance genes (R genes) which support the prior observation that many barley resistance genes are located distally in regions with high recombination frequency (International Barley Genome Sequencing Consortium [IBSC], 2012). It has been indicated, that more than 80% of all known R genes are of the NBS-LRR type (nucleotide-binding leucine rich repeat) (Shao et al., 2016). LRR domains have particular function in plant-pathogen recognition (Hong and Zhang, 2016). The annotation using Blastx against the non-redundant protein database of NCBI also indicates the presence of the NBS-LRR domain in all five disease resistance genes in the target interval. Disease resistance genes located in the target interval tend to cluster which is typical for NBS-LRR based resistance gene analogs (DeYoung and Innes, 2006). Since *P. hordei* is a biotrophic fungi and the fact that NBS-LRR resistance genes are only effective in conferring resistance to biotrophic or hemibiotrophic pathogens, but not against necrotrophic pathogens (Belkhadir et al., 2004) provides evidence that resistance is due to a gene carrying the NBS-LRR motif. Hence, full length re-sequencing of five disease resistance genes in parental lines was conducted. However, more than 80% similarity in the sequences of R genes considerably hampered sequencing, therefore in order to obtain a complete sequence of the disease resistance genes, new primer design will be conducted.

Marker validation of seven co-segregating markers in 51 already tested barley lines (König et al., 2012), as well as 12 other barley cultivars/lines, gave hint that new markers identified in this study are not all diagnostic for *Rph*_*MBR*1012_. Based on our study, the markers GBS546 and GBS626 with 80.32 and 83.60% accuracies in prediction of *Rph*_*MBR*1012_ are the best diagnostic markers and facilitate faster and easier detection of *Rph*_*MBR*1012_ (and putative alleles) in barley breeding lines. Out of the selected markers QBS128 (HORVU1Hr1G000830/Disease resistance protein), QBS130 (HORVU1Hr1G000910/Disease resistance protein), QBS116, QBS117 and GBR534 (HORVU1Hr1G000940/copper ion binding), and marker GBS546 (HORVU1Hr1G000930/Low molecular weight glutenin subunit) were directly derived from putative candidate genes in the target interval but revealed a less diagnostic character. However, the diagnostic *Rph*_*MBR*1012_ markers identified in this study could be very useful not only for discriminating between resistant and susceptible cultivars but also for pyramiding of *Rph*_*MBR*1012_ with other resistance genes to aim a durable resistance in barley cultivars (Sharma Poudel et al., 2018).

## Conclusion

In summary, by using high-throughput genotyping and sequencing techniques together with the barley reference sequence we succeeded to downsize the *Rph*_*MBR*1012_ target interval to 0.44 Mb between markers QBS127 and QBS98 in comparison to 6.24 Mb in a previous study. This is an indispensable step toward isolation of this gene. Four strategies might be then considered in next step in order to define the loci underlying the resistance gene *Rph*_*MBR*1012_; enhancing the map resolution via screening a new set of F_2_ plants and using the new SNPs and InDel defined from candidate genes at target interval to develop the new markers for further marker saturation, screening a non-gridded BAC library from donor line MBR1012, overexpression of five detected disease resistance genes in the target interval in a susceptible barley cultivar, e.g., Scarlett and knock out the genes in resistant lines using CRISPR/cas9. The co-segregating and closely linked markers detected in this study, may be useful as probes for BAC library screening and construction of the physical map in MBR1012.

## Author Contributions

DP, DK, and FO conceived and designed the experiments, provided the experimental material and contributed to study design, subject recruitment and sample preparation. LF, DK, and DP performed the experiments. LF, JK, and DP analyzed the data. LF, JK, HD, FO, and DP interpreted the data. All authors wrote the manuscript, and read and approved the final manuscript.

## Conflict of Interest Statement

The authors declare that the research was conducted in the absence of any commercial or financial relationships that could be construed as a potential conflict of interest.

## Footnotes

1 http://bioinfo.ut.ee/primer3-0.4.0/

2 http://www.lgcgroup.com/

3 http://tools.neb.com/NEBcutter2

4 http://webblast.ipk-gatersleben.de/barley_ibsc/

5 https://plants.ensembl.org/Hordeum_vulgare/Info/Index

6 https://www.ncbi.nlm.nih.gov/sutils/splign/splign.cgi?textpage=online&level=form
